# Analysis of the Urine

**Published:** 1877-06

**Authors:** 


*Chemical and Microscopical Analysis of the Urine in Health and Disease.* Designed for Physicians and Students by Geo. B. Fowler, M. D., Examiner in Physiology, College of Physicians and Surgeons, New York, Visiting Surgeon to the New York Dispensary, etc. Second Edition, Revised and Enlarged, with Eighteen Illustrations. New York: G. P. Putnam's Sons, 182 Fifth Avenue, 1876, pp. 97, price $1.00. Buffalo, Peter Paul & Bro.

The author says, "In this as in the first edition the object has been to present the most practical and important features of the subject."

His aim is realized, and the result is a very convenient monograph of this important feature of a physician's work. The proper lens power for microscopical examinations is given, also a list of the required apparatus and reagents for chemical analysis, and their cost.

Precautions to be observed with regard to reactions are enforced and comprehensive general directions are added to the detailed instructions which make up the body of the book.

Works of this sort which render diagnosis easier and more accurate cannot be too warmly commended.

